# Replacing Dietary Roughage with Barley Hay Affects Rumen Fermentation, Microbial Community, Serum Immune and Antioxidant Status in Sheep

**DOI:** 10.3390/ani16101565

**Published:** 2026-05-21

**Authors:** Xiaoyuan Wang, Xinyi Liu, Lahan Hai, Guoli Han, Khas Erdene, Chen Bai, Qina Cao, Yankai Zheng, Zhiping Liu, Changjin Ao

**Affiliations:** 1 Key Laboratory of Animal Nutrition and Feed Science, College of Animal Science, Inner Mongolia Agricultural University, Zhaowuda Road 306, Saihan District, Hohhot 010008, China; 2 Institute of Crop Science, Inner Mongolia Academy of Agriculture and Animal Husbandry Sciences, Hohhot 010018, China

**Keywords:** barley hay, high-throughput, rumen ecology, blood, lambs

## Abstract

Barley hay (**BH**), characterized by high crude protein, neutral detergent fiber, and various bioactive compounds, is hypothesized to optimize ruminal microbiota and enhance systemic immune responses in sheep. This study assessed how the graded substitution of traditional forage with BH impacts ruminal fermentation, microbiota composition, and blood immune and antioxidant status in sheep. Forty-five male Dorper × Small-tailed Han crossbred lambs (aged 110 ± 10 days, BW 33.93 ± 1.11 kg) were stratified into five cohorts (*n* = 9 each), including a control (CON) and four experimental groups (BH25, BH50, BH75, and BH100), where BH replaced 25%, 50%, 75%, and 100% of their forage mixture, respectively. Results showed that BH supplementation consistently increased ruminal acetate concentrations. Specifically, BH75 and BH100 significantly enhanced the acetate-to-propionate ratio (*p* < 0.05) and concurrently reduced NH_3_-N concentrations in BH50 and BH100 (*p* < 0.05). Regarding ruminal microbiota, BH treatment did not alter alpha diversity but significantly enriched fiber-degrading bacteria, including *Clostridia*, *Bacteroidales*, and *Prevotella*, whereas the CON group favored *Eubacterium*. Blood analysis indicated a time-dependent modulation of immune and antioxidant markers. At day 30, BH treatment resulted in elevated immunoglobulin A (IgA), immunoglobulin G (IgG), interleukin-6 (IL-6), and malondialdehyde (MDA) levels (*p* < 0.05), whereas catalase (CAT) concentrations were markedly reduced (*p* < 0.05). By day 60, BH continued to enhance IgA, IgG, and IL-6 levels; meanwhile, tumor necrosis factor α (TNF-α) levels were up-regulated by BH25-BH75 (*p* < 0.05), while supplementation with BH75 and BH100 led to a decline in interleukin-4 (IL-4), CAT, and interleukin-10 (IL-10) (*p* < 0.05). Correlation analysis showed that a positive correlation was observed between total volatile fatty acids and *Prevotella UCG-003/001*, which conversely displayed a negative relationship with IgM. The *norank f Eubacterium coprostanoligenes group* showed an inverse association with valerate and IgG, but a positive correlation with interleukin-1β (IL-1β). In parallel, the *norank f Bacteroidales RF16 group* exhibited an inverse association with NH_3_-N and CAT, while *unclassified c Clostridia* was positively correlated with IL-1β. In conclusion, BH substitution modulates ruminal fermentation and immune responses by enriching fiber-degrading bacteria, though it may trigger oxidative stress. Given that BH100 maximized acetate and immunoglobulin production while minimizing MDA levels, complete replacement appears superior to partial substitution.

## Simple Summary

Feeding sheep traditional forage, such as corn stalks, often provides limited nutritional value, which can hinder the animals’ growth and immune health. This study investigated whether replacing these traditional feeds with barley hay could improve sheep health. We fed forty-five young sheep diets where barley hay replaced 25%, 50%, 75%, and 100% of their usual roughage. Our findings revealed that barley hay is an excellent dietary choice because it facilitates the proliferation of fiber-degrading microbes within the sheep gut, which enhances feed digestion efficiency. Furthermore, this diet enhanced natural immunity, which was confirmed by increased concentrations of protective serum proteins. Although adjusting to new feed can sometimes cause minor stress to the body, feeding the sheep a diet consisting entirely of barley hay proved to be the most effective strategy. This approach significantly improved the sheep’s digestion and immune strength without causing lasting health issues. Using barley hay as a primary feed source offers a practical and natural way for farmers to enhance the overall health and productivity of their livestock, ultimately contributing to more sustainable sheep farming practices.

## 1. Introduction

The rapid development of global sheep farming is currently hampered by a critical scarcity of premium-quality roughage. While conventional roughages such as corn stover and *Leymus chinensis* are widely utilized due to their low cost, they are inherently limited by poor crude protein content and high lignification [1]. These nutritional deficiencies impede rumen microbial degradation efficiency, ultimately compromising the growth performance of the animals [2,3]. Furthermore, mutton sheep during the intensive fattening phase face heightened metabolic demands, making them particularly vulnerable to oxidative stress and metabolic disorders if the nutrient supply is inadequate [4]. Consequently, identifying sustainable alternative roughages capable of simultaneously optimizing rumen fermentation and safeguarding host health remains a paramount objective in modern ruminant nutrition.

Barley (*Hordeum vulgare* L.) is receiving increasing attention as a promising alternative feed source. Unlike conventional feedstuffs like cereal straw and ryegrass, whole-plant barley hay offers a superior crude protein level and a more balanced neutral detergent fiber (NDF) composition. In addition, it is rich in key bioactive compounds, especially β-glucans and plant polyphenols [5,6]. The β-glucans in barley are unique water-soluble dietary fibers that resist degradation in the upper digestive tract and act as prebiotics—either reaching the hindgut or being fermented by specific microbes in the rumen [7,8]. Its abundant polyphenols, including flavonoids and phenolic acids, contribute to barley’s biological potential [9,10,11]. Previous studies have shown that β-glucans can serve as effective immune modulators by stimulating macrophages and lymphocytes, thereby activating both mucosal and systemic immune responses [12,13]. Meanwhile, plant-derived polyphenols or their extracts can significantly improve oxidative status and stress resilience in animals, ultimately benefiting growth and health [14,15]. For ruminants, feeding barley-based diets can modify the rumen environment and enhance fiber digestibility [16]. However, a comprehensive evaluation of how sustained substitution of traditional roughages with barley hay (BH) impacts the intricate interplay between the ruminal microbiota, host immune responses, and oxidative health remains to be elucidated.

The rumen microbiome serves as a central hub driving host metabolism in ruminants, as their physiological responses largely depend on the extent to which these microbes ferment dietary substrates. Dietary fiber profile serves as a key determinant in sculpting the composition of the ruminal microbial ecosystem [17]. Consequently, the specific fiber profile of barley hay contains a relatively high proportion of readily fermentable carbohydrates, which may selectively enrich certain fiber-degrading bacteria, such as *Bacteroidota* and *Firmicutes* [18], thereby altering the profile of volatile fatty acids (VFA) [19]. These key metabolic products could, in turn, further influence systemic immune function and antioxidant status in meat sheep [20,21].

Therefore, we hypothesize that replacing traditional roughage with BH will reshape the rumen microbiota, optimize fermentation efficiency, and consequently modulate host immunity and antioxidant status in mutton sheep. Accordingly, we evaluated how graded substitution of BH at 0%, 25%, 50%, 75%, and 100% levels replacing the basal roughage on rumen fermentation parameters, core rumen microbiota, and serum immune and antioxidant markers in meat sheep. We anticipate that these findings will establish a theoretical framework for utilizing barley hay within ruminant diets to balance metabolic efficiency and host health.

## 2. Materials and Methods

### 2.1. Ethics Statement

All animal procedures in this trial strictly followed the “Guiding Opinions on the Humane Treatment of Laboratory Animals” (Ministry of Science and Technology of China, 2006).

### 2.2. Study Site and Material Preparation

The trial took place between November 2023 and January 2024 at Fuchuan Husbandry Technology Co., Ltd. (Bayannur, Inner Mongolia, China). The Mengpimay No. 5 barley hay used was supplied by the Inner Mongolia Academy of Agricultural and Animal Husbandry Sciences.

### 2.3. Experimental Design and Animal Management

Forty-five healthy, weaned Dorper × Small-tailed Han crossbred male lambs (110 ± 10 days; BW = 33.93 ± 1.11 kg) were allocated into five experimental cohorts. Each cohort consisted of three replicate pens, each housing three lambs. Lambs were group-housed and group-fed within their respective pens. This experimental design, comprising 9 individual sheep per treatment group (distributed across 3 replicate pens), strictly complies with biostatistical principles. It ensures adequate biological replication and statistical power, particularly for accounting for individual variations in the downstream rumen microbiome analyses. The control (CON) received a basal diet, while the other four cohorts (BH25, BH50, BH75, and BH100) received diets with 25%, 50%, 75%, and 100% of the forage replaced by BH, respectively. To strictly isolate the specific effects of BH and eliminate confounding factors from dietary variations, all experimental diets were meticulously formulated to be isocaloric and isonitrogenous. Consequently, the varying inclusion level of BH was maintained as the sole independent variable across the experimental groups. The 75-day study involved a 15-day adaptation phase, followed by a 60-day experimental recording phase. Lambs were kept in indoor facilities with daily hygiene maintenance and constant access to clean water. Total mixed rations (TMR) were provided twice daily at 08:00 and 16:00. Dietary formulation was designed to align with the Nutrient Requirements of Mutton Sheep (NY/T 816-2021) [22] while accounting for regional feeding practices. Comprehensive details regarding ingredient and nutrient composition are provided in Table 1 and Table 2.

### 2.4. Sample Collection

#### 2.4.1. Feed Sampling

Feed samples were routinely gathered throughout the experiment and kept at −20 °C until chemical analysis.

#### 2.4.2. Rumen Fluid Collection

At the experiment’s conclusion, rumen fluid was obtained from each lamb using an oral stomach tube fitted with a metal strainer (A1164K, Wuhan Anscitech Farming Technology Co., Ltd., Wuhan, China). After discarding the initial 100 mL to prevent salivary contamination, we collected 200 mL of ruminal fluid per animal. A sub-sample was instantly snap-frozen in liquid nitrogen for future microbial analysis. The rest was filtered using four layers of cheesecloth. Filtrate pH was recorded immediately using a portable meter (PHS-3C, Shanghai Osterol Industrial Co., Shanghai, China). Finally, the remaining filtered rumen fluid was aliquoted and frozen in liquid nitrogen for subsequent ammonia nitrogen (NH_3_-N) and VFA quantification.

#### 2.4.3. Serum Sampling

On experimental days 30 and 60, lambs underwent a 12 h overnight fast with water deprivation. Before morning feeding, 10 mL of blood was collected from the jugular vein into non-anticoagulant vacuum tubes (Huabo Medical Instruments Co., Ltd., Nanjing, China). Samples were kept at room temperature for 40 min to allow clotting, then centrifuged (13,000× *g*, 10 min) for serum separation. The serum was transferred into Eppendorf tubes and stored at −20 °C until antioxidant capacity evaluation.

### 2.5. Chemical and Biological Analyses

#### 2.5.1. Feed Chemical Composition

Dried and ground feed samples were analyzed for chemical composition. Contents of CP, Ca, and P were measured following the protocols established by the AOAC [24] (CP: method 990.03; Ca: 968.05; P: 975.16). Fiber fractions (NDF and ADF) were analyzed using an ANKOM A200i system (ANKOM Technology, Macedon, NY, USA) according to standard methods 2002.04 and 973.19.

#### 2.5.2. Rumen Fermentation Parameters

Ruminal NH_3_-N levels were measured via the colorimetric assay of Broderick and Kang. VFA profiles were analyzed according to Matra et al. Briefly, VFA concentrations were quantified via gas chromatography (GC-2014, Shimadzu, Kyoto, Japan) with a flame ionization detector (FID) and a capillary column (60 m × 0.25 mm × 0.50 μm). Analytical conditions were optimized: nitrogen was used as carrier gas (1.74 mL/min; linear velocity: 32.6 cm/s; split ratio: 40:1). The oven temperature was 180 °C, the injection port was 220 °C, and the detector was 250 °C. Gas flow rates were adjusted to 55, 40, and 3 mL/min for hydrogen, air, and makeup gas, respectively.

#### 2.5.3. Rumen Microbiota Sequencing

To facilitate cell lysis, rumen fluid samples underwent lyophilization and grinding. We isolated genomic DNA utilizing the E.Z.N.A.^®^ Soil DNA Kit (Omega Bio-tek, Norcross, GA, USA), strictly adhering to the manufacturer’s manual. DNA purity and yield were evaluated through 1% agarose gel electrophoresis and NanoDrop 2000 spectrophotometry. Primers 338F (5′-ACTCCTACGGGAGGCAGCAG-3′) and 806R (5′-GGACTACHVGGGTWTCTAAT-3′) were employed to amplify the bacterial 16S rRNA gene (V3–V4 region). PCR thermal cycling comprised an initial denaturation at 95 °C for 3 min, followed by 27 cycles (95 °C for 30 s, 55 °C for 30 s, and 72 °C for 30 s), and a final extension at 72 °C for 10 min. Amplicons were verified by gel electrophoresis, excised, and purified. Subsequently, sequencing was performed on the Illumina MiSeq PE250 platform (Majorbio, Shanghai, China). Raw sequences were processed via the QIIME2 pipeline to generate ASVs, and taxonomic classification was conducted referencing the Silva 138 database.

#### 2.5.4. Serum Immune and Antioxidant Parameters

We measured serum immune and antioxidant markers utilizing commercial ELISA kits (Jiangsu Meimian Industrial Co., Ltd., Yancheng, China) following the provided protocols. Immune markers quantified included immunoglobulin levels (IgA, IgG, IgM), interleukins (IL-1β, IL-4, IL-6, IL-10), and tumor necrosis factor-α (TNF-α). Antioxidant metrics determined were total antioxidant capacity (T-AOC), superoxide dismutase (SOD), catalase (CAT), glutathione peroxidase (GSH-Px), and malondialdehyde (MDA) content.

### 2.6. Data Analysis

Data normality was checked via the Shapiro–Wilk test before statistical processing. We performed one-way ANOVA on rumen fermentation and serum parameters using SAS 9.21 (SAS Institute Inc., Raleigh, NC, USA), with Duncan’s multiple range test for post hoc comparisons. Significance was defined at *p* < 0.05, high significance at *p* < 0.01, and a statistical trend at 0.05 ≤ *p* < 0.10.

## 3. Results

### 3.1. Effects of Dietary Barley Hay Proportion on Rumen Fermentation in Lambs

The impact of barley hay substitution on ruminal fermentation is summarized in Table 3. All barley hay treatments significantly increased acetate concentration (*p* < 0.05) compared with the control, with no significant differences among treatment groups. Similarly, the acetate to propionate (A:P) ratio was elevated across all substitution levels, peaking in the BH75 group (*p* < 0.05). Additionally, all treated lambs exhibited lower NH_3_-N levels than the control, with the lowest values observed in the BH50 group (*p* < 0.05).

### 3.2. Microbial Community Composition

**Figure 1:**
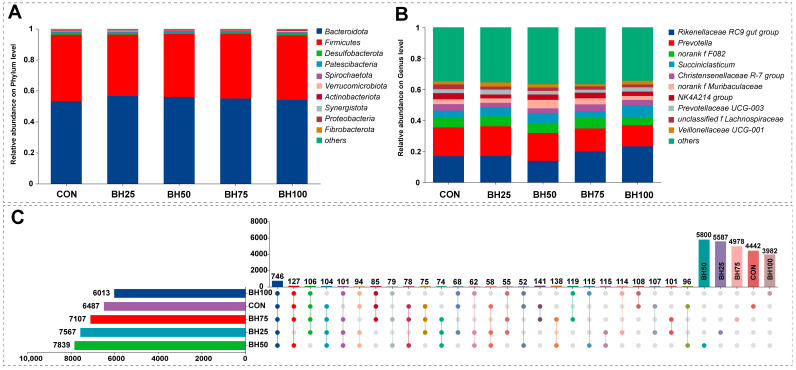
**Characterization of Rumen Microbial Composition**. (**A**,**B**) Stacked bar plots of dominant microbial taxa at phylum (**A**) and genus levels (**B**). The *X*-axis indicates samples, while the *Y*-axis denotes relative abundance. Colors signify distinct taxa, with segment height reflecting their respective proportions. (**C**) UpSet plot of microbial communities. The left horizontal bars show total taxon counts per group. In the matrix, isolated dots denote unique taxa, whereas connected dots signify intersections between groups. The vertical bars above the matrix represent the number of taxa within each corresponding intersection. BH25, BH50, BH75, and BH100 denote barley hay substitution levels of 25%, 50%, 75%, and 100%, respectively.

Figure 1 displays the composition of the ruminal microbiota. At the phylum level (Figure 1A), Bacteroidota and Firmicutes were dominant, collectively exceeding 90% in all groups, followed by Desulfobacterota, Patescibacteria, Spirochaetota, Verrucomicrobiota, Actinobacteriota, Synergistota, Proteobacteria, and Fibrobacterota.

Among the top 10 genera (Figure 1B), microbial composition was similar across groups. Dominant genera, ranked by relative abundance, included *Rikenellaceae RC9 gut group*, *Prevotella*, *norank f F082*, *Succiniclasticum*, *Christensenellaceae R-7 group*, *norank f Muribaculaceae*, *NK4A214 group*, *Prevotellaceae UCG-003*, *unclassified f Lachnospiraceae*, and *Veillonellaceae UCG-001*.

Figure 1C (UpSet plot) details the distribution of taxa beyond the top 10. Total taxon counts across groups were as follows: 7839 (BH50), 7567 (BH25), 7107 (BH75), 6487 (CON), and 6013 (BH100). Regarding unique taxa, BH50 had the highest count (5800), followed by BH25 (5587), BH75 (4978), CON (4442), and BH100 (3982). Furthermore, intersection analysis identified 746 taxa common to both CON and all BH-treated groups (core microbiota) and 101 taxa unique to all BH treatment groups. Pairwise shared taxa between CON and specific BH groups were 141 (BH75), 108 (BH100), 107 (BH25), and 50 (BH50).

### 3.3. Community Diversity Analysis

**Figure 2:**
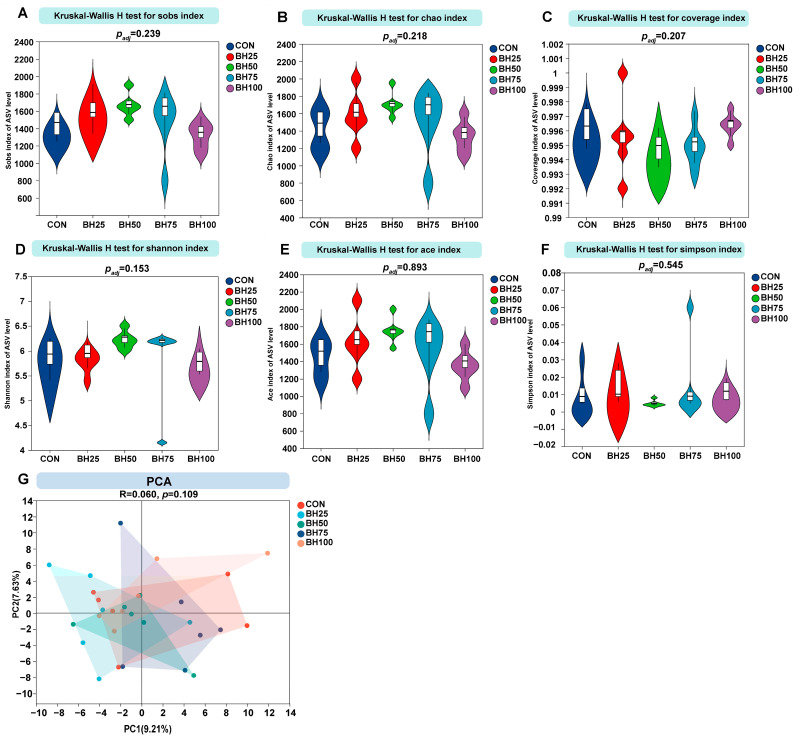
**Rumen microbial diversity analysis.** (**A**–**F**) Alpha diversity indices: Sobs (**A**), Chao (**B**), Coverage (**C**), Shannon (**D**), Ace (**E**), and Simpson (**F**). The *X*-axis displays groups, while the *Y*-axis shows corresponding diversity indices. (**G**) Beta diversity via PCA. Axes denote principal components (PC1, PC2) with variance contribution percentages. Samples are differentiated by color and shape; proximity between points indicates similarity in microbial community composition. BH25–BH100 represent barley hay inclusion levels of 25–100%.

Table 4 and Figure 2A–F summarize the alpha diversity response to dietary barley hay substitution. As shown in Table 4, the richness estimators, including Sobs, Chao, and Ace, exhibited a non-linear response to increasing BH inclusion levels. Numerically, the highest species richness was observed in the BH50 group, where Sobs, Chao, and Ace index reached their peak compared to the CON and other BH treatment groups. However, Kruskal–Wallis H tests indicated these differences were not significant (Adjusted *p* > 0.05). The violin plots (Figure 2A,B,E) further illustrate the substantial overlap in distribution and median values across all BH treatment groups. The Shannon diversity index (Figure 2D) showed a slight upward trend in the BH group compared to the CON group, but the overall difference remained non-significant. Similarly, the Simpson index (Figure 2F), which measures evenness, remained stable across all treatments. The Coverage index (Figure 2C) remained consistently at 1.00 for all samples across the five groups. Similar coverage indices confirmed that sequencing depth was sufficient to capture the vast majority of the microbial taxa present in the samples.

We evaluated overall microbial community structure via ASV-level Principal Component Analysis (PCA) (Figure 2G). The first two principal components, PC1 and PC2, explained 9.21% and 7.63% of the total variance, respectively, accounting for a cumulative total of 16.84%. Visual inspection of the PCA plot reveals a substantial overlap among the convex hulls of all groups, with individual sample points from different treatments interspersed without forming distinct, isolated clusters. Statistical analysis supported these visual observations, yielding an R-value of 0.060 and a *p*-value of 0.109. Since the *p*-value exceeded the standard significance threshold (*p* > 0.05), this suggests that barley hay substitution did not significantly alter the global microbial community structure of the microbial community, suggesting a high degree of stability in the gastrointestinal ecosystem across all treatment levels.

### 3.4. Differential Abundance Analysis

**Figure 3:**
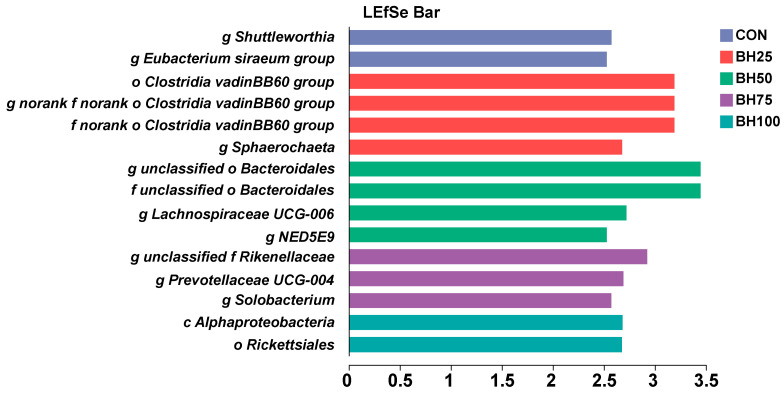
**Linear Discriminant Analysis (LDA) of differentially abundant taxa**. The bar plot displays taxa with significant inter-group differences identified *via* LEfSe. LDA scores (log_10_) quantify the contribution of specific taxa to inter-group dissimilarities; higher scores indicate a more substantial influence on community differences.

We used LEfSe to identify microbial taxa significantly enriched in each group (LDA > 2.0; Figure 3). Across the five groups, 15 biomarkers were identified. Specifically, the CON group was enriched in the *Shuttleworthia* and *Eubacterium siraeum* groups. *Biomarkers* in the BH25 group primarily belonged to the *Clostridia vadinBB60* group and *Sphaerochaeta*. The BH50 group showed enrichment in *Bacteroidales*, *Lachnospiraceae UCG-006*, and *NED5E9*. The BH75 group was enriched in *Rikenellaceae*, *Prevotellaceae UCG-004*, and *Solobacterium*, while the BH100 group was characterized by *Alphaproteobacteria* and *Rickettsiales*.

### 3.5. Effects of Dietary Barley Hay Proportion on Serum Immune Levels in Lambs

As indicated in Table 5, all BH inclusion groups displayed elevated IgA concentrations on day 30 (*p* < 0.001). Relative to CON, IgG levels significantly peaked in BH50 and BH75 (*p* < 0.001). IL-4 increased in BH25 and BH50 (*p* < 0.05) but remained stable in BH75 and BH100. BH inclusion uniformly increased IL-6, with BH50 and BH75 recording higher levels than BH25 and BH100 (*p* < 0.001). Additionally, the BH100 group reached the highest IL-10 levels across all groups (*p* < 0.05).

On day 60, IgA concentrations peaked in the BH75 group, with CON registering the lowest levels (*p* < 0.001). IgG levels in BH50 and BH75 significantly exceeded those in CON, BH25, and BH100 (*p* < 0.05), while BH100 showed no significant differences from CON. Regarding IL-4, levels were significantly higher in BH75 and BH100 than in other groups (*p* < 0.001). IL-6 levels in all BH treatments surpassed CON, with the highest values observed in the BH75 group. Finally, all BH treatments except BH100 induced higher TNF-α concentrations compared to CON (*p* < 0.001).

### 3.6. Effects of Dietary Barley Hay Proportion on Serum Antioxidant Levels in Lambs

Table 6 displays the impact of BH inclusion on serum antioxidant status. On day 30, serum CAT levels in all BH treatment groups were significantly lower than those in the CON group (*p* < 0.05). Conversely, serum MDA levels were significantly elevated across all BH groups compared with CON (*p* < 0.05).

On day 60, a similar trend was observed for CAT; BH inclusion consistently decreased CAT levels relative to CON, with the lowest value recorded in the BH25 group (*p* < 0.001). Furthermore, BH75 exhibited higher serum MDA than both CON and BH25 groups (*p* < 0.05).

### 3.7. Impacts of Key Rumen Microbiota on Fermentation Parameters, Serum Immunity, and Antioxidant Indices

**Figure 4:**
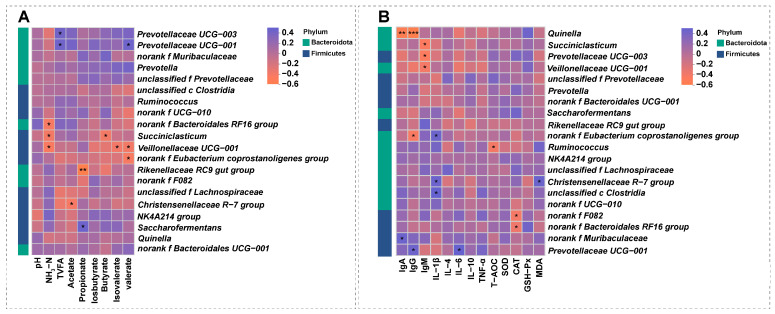
**Correlation analysis of key rumen microbiota and physiological parameters**. (**A**) Heatmap showing the correlations between key rumen microbial taxa and fermentation parameters. (**B**) Heatmap showing the correlations between key rumen microbial taxa and blood immune and antioxidant indices. The *X*-axis and *Y*-axis represent fermentation/serum parameters and microbial taxa, respectively. The color gradient indicates the Spearman correlation coefficient (R-value), with the scale shown in the legend on the right. Statistical significance is indicated by asterisks: *, ** and *** indicate *p* ≤ 0.05, *p* ≤ 0.01 and *p* ≤ 0.001, respectively.

Spearman correlation heatmaps were generated to evaluate the associations between the top 20 microbial genera and ruminal fermentation parameters (Figure 4A). *Prevotellaceae UCG-003* was positively correlated with TVFA. *Prevotellaceae UCG-001* was positively correlated with TVFA and valerate. Saccharofermentans was positively correlated with acetate. In contrast, *norank f Bacteroidales RF16 group*, *Succiniclasticum*, and *Veillonellaceae UCG-001* were negatively correlated with NH_3_-N. The *Christensenellaceae R-7 group* was negatively correlated with acetate. The *Rikenellaceae RC9 gut group* was highly negatively correlated with propionate. *Succiniclasticum* was negatively correlated with butyrate. *Veillonellaceae UCG-001* was negatively correlated with isovalerate and valerate. The *norank f Eubacterium coprostanoligenes group* was negatively correlated with valerate.

As shown in Figure 4B, the correlations between serum immune and antioxidant indices and the rumen top 20 microbial communities showed several notable associations. Among the immunoglobulins, IgA was significantly negatively correlated with *Quinella* but positively correlated with *norank f Muribaculaceae*. Similarly, IgG showed negative correlations with both *Quinella* and *norank f Eubacterium coprostanoligenes group*, but was positively correlated with *Prevotella UCG-001*. In contrast, IgM had negative correlations with *Succiniclasticum*, *Prevotellaceae UCG-003*, and *Veillonellaceae UCG-001*, while showing positive correlations with *norank f Eubacterium coprostanoligenes group*, *Christensenellaceae R-7 group*, and *unclassified c Clostridia*. For the inflammatory marker, IL-6 was positively correlated with *Prevotellaceae UCG-001*. Regarding the antioxidant indices, T-AOC was negatively correlated with *Ruminococcus*, and CAT showed negative correlations with both *norank f F082 and norank f Muribaculaceae*. Notably, MDA, an indicator of oxidative stress, was positively correlated with the *Christensenellaceae R-7 group*.

## 4. Discussion

### 4.1. Impact of Barley Hay Substitution on Ruminal Fermentation and Nitrogen Efficiency

Ruminal fermentation parameters are indicators of the host’s metabolic status and the efficiency of dietary energy utilization [25,26]. In this study, the substitution of conventional forage (corn stover and *Leymus chinensis*) with BH significantly reshaped the VFA profile, most notably by increasing acetate concentrations and the acetate-to-propionate ratio. This shift is likely attributable to the unique physicochemical structure of the BH fiber [27]. Unlike the highly lignified cellulose found in corn stover, BH contains a higher proportion of hemicellulose and pectin, which are more accessible to microbial cellulolytic enzymes [28]. According to Beauchemin [29], the fermentation of these specific structural carbohydrates inherently favors the acetogenic pathway. Our findings align with Guo et al. [30], who demonstrated that replacing low-quality roughage with high-quality forage enhances TVFA production, thereby increasing the supply of metabolizable energy for host growth and lactation.

In this study, a notable finding was the significant reduction in NH_3_-N levels in the BH groups, with the BH50 group reaching the lowest concentrations. In the rumen, NH_3_-N concentration is the net result of dietary protein degradation and microbial nitrogen uptake [31]. The reduction observed here is consistent with the “energy-nitrogen synchronization” theory. As proposed by Brassard et al. [32,33,34], when the degradation rate of carbohydrates matches the release of nitrogen from dietary protein, microbial protein synthesis is maximized, leaving less residual ammonia in the rumen fluid. The fermentable carbohydrates in BH, particularly the water-soluble sugars and non-starch polysaccharides, likely provided the immediate energy required by microbes to incorporate ammonia into microbial biomass [35,36]. This mechanism is crucial for improving nitrogen retention efficiency, a key goal in sustainable ruminant production to reduce environmental nitrogen excretion [37]. Furthermore, the stability of ruminal pH across all groups, despite higher VFA production, suggests that BH substitution maintains a robust buffering capacity. As noted by Nocek and Russell [38,39], the physical effectiveness of fiber in barley hay may stimulate rumination and saliva secretion, which counteract the acidifying effect of rapid fermentation.

### 4.2. Microbiota Remodeling as a Driver of Altered Metabolic Pathways

The shifts in fermentation products are closely associated with the structural and functional restructuring of the ruminal microbial community [40]. Our 16S rRNA sequencing data revealed that BH substitution did not merely alter the abundance of core phyla but specifically enriched functional genera known to be involved in complex fiber degradation [41]. For instance, the significant enrichment of *Prevotellaceae UCG-003* and *Saccharofermentans* in the BH groups is a key finding. *Prevotellaceae* are renowned for their high genetic plasticity and diverse enzymatic repertoire, allowing them to degrade a wide range of non-cellulosic polysaccharides such as hemicellulose and pectin [42,43]. In the context of BH inclusion, which provides a distinct matrix of structural and readily fermentable carbohydrates, the enrichment of these taxa highlights a targeted microbial adaptation [44]. Previous studies suggest that *Prevotellaceae UCG-003* acts as a primary degrader of the plant cell wall integrity [45], which may increase the accessibility of downstream substrates for secondary fermenters like *Saccharofermentans* [44,45,46,47]. By accelerating the ruminal turnover rate of these liberated carbohydrates, this targeted microbial consortium efficiently maximizes the conversion of dietary carbon into the ruminal VFA pool [48]. Consequently, the positive correlation between these enriched taxa and TVFA production supports their potential role as primary drivers of enhanced energy extraction specifically adapted to the BH-based diet.

The LEfSe analysis elucidated a distinct successional trajectory of the ruminal microbiome driven by the progressive substitution of highly lignified roughage with highly digestible BH [49]. In the BH50 group, the pronounced enrichment of *Bacteroidales* and *Lachnospiraceae UCG-006* suggests an adaptive expansion of functionally versatile degraders targeting moderately degradable plant cell wall polymers, a hallmark of a mature and efficient fibrolytic ecosystem [50]. As the BH inclusion rate increased (BH75), the microbial community pivoted towards a highly specialized consortium characterized by the co-enrichment of *Rikenellaceae* and *Prevotellaceae UCG-004* [51]. Notably, the *Rikenellaceae RC9 gut group*, a key biomarker identified in our study, exhibited a robust negative correlation with ruminal propionate and a strong positive association with the acetate-to-propionate ratio [52]. This distinct correlative pattern provides a mechanistic explanation for the observed fermentation shift: rather than merely suppressing the propionate pathway, the high-quality, readily fermentable fiber in BH predominantly stimulates the proliferation of *Rikenellaceae*, thereby promoting the acetate-yielding pathways [53]. Furthermore, the concurrent shifts in taxa such as *Veillonellaceae* and *Succiniclasticum*, which participate in converting succinate to propionate [54,55], indicate that BH substitution appears to modulate the complex cross-feeding networks within the rumen. This metabolic rewiring transforms the rumen fermentation pattern from a slow-degrading state typical of recalcitrant stover to a highly dynamic, fiber-focused, and acetate-dominant system. This shift closely corresponds to the thermodynamic changes observed in hydrogen-utilizing pathways during the transition to high-quality forage diets [56,57].

### 4.3. Linking Ruminal Changes to Host Immunity and Antioxidant Status

The modulation of the rumen ecosystem by BH substitution exerts a notable influence on the systemic physiological health of the animal, highlighting a critical “rumen-blood” axis of regulation [58]. The introduction of highly fermentable forage is generally presumed to be beneficial, and our results reflect this through enhanced humoral immune markers, specifically the elevations in IgA and IgG [59]. This immune-enhancing effect can be partially attributed to bioactive compounds naturally abundant in barley, such as β-glucans and specific polyphenols [60]. As biological response modifiers, β-glucans can bind to Dectin-1 receptors on intestinal immune cells, triggering a cascade that enhances humoral immunity [61]. This is further augmented by the altered microbiome, where robust positive correlations between *Prevotellaceae UCG-003* and *Prevotellaceae UCG-001* and TVFA imply their involvement in active fermentation [62]. The resulting influx of VFAs into the bloodstream likely acts *via* G-protein-coupled receptors, such as GPR41 and GPR43, to further prime the gut-associated lymphoid tissue (GALT) [63,64,65]. However, specific microbial shifts act as nuanced regulators rather than simple enhancers; for instance, the rapid proliferation of *Quinella*—often a signature of high-efficiency fermentation [66]—exhibited a significant negative correlation with IgA and IgG, suggesting that its excessive expansion might be related to shifts in specific mucosal immune pathways, though the exact mechanisms require further investigation [67].

Despite these immune benefits, our results revealed a paradoxical effect dependent on the substitution dose concerning host physiological homeostasis. While specific immunological barriers (IgA, IgG) were strengthened, an evident perturbation involving both oxidative stress and mild systemic inflammation was observed. This was evidenced by a significant decline in serum CAT activity and a concomitant elevation in MDA levels, coupled with notable increases in the pro-inflammatory cytokines IL-6 and TNF-α, particularly in groups with elevated inclusion of BH.

This coupled phenomenon, often identified as metabolic oxidative burden [68], may be associated with specific underlying mechanisms. The superior degradability and rapid fermentation rate of BH could potentially facilitate a surge in nutrient flux to the liver and peripheral tissues [69]. As cellular mitochondria accelerate ATP synthesis to match this heightened metabolic rate, this process is likely to be accompanied by a concurrent transient rise in reactive oxygen species (ROS) leakage. The excessive ROS not only may potentially challenge the endogenous antioxidant defense system [70,71], but also serves as a potent signaling trigger that activates inflammatory cascades, thereby potentially stimulating the release of IL-6 and TNF-α.

The correlation analysis suggests a potential mechanistic link. While the *Christensenellaceae R-7 group* was positively correlated with MDA, which may imply its possible association with the robust energy metabolism that generates reactive oxygen species [72,73], it simultaneously exhibited a negative correlation with pathways promoting inflammation. *Christensenellaceae* are widely regarded as markers of health [74], and their enrichment could potentially contribute to mitigating the systemic subclinical inflammation that is frequently associated with oxidative stress and diets dense in energy [75].

### 4.4. Limitations and Future Perspectives

Although the findings of this study provide important insights into the effects of replacing BH, the current research has some limitations. The short trial period may limit our ability to assess long-term changes in the rumen ecosystem. Because ruminants have high metabolic plasticity, the lack of long-term data means we cannot yet confirm whether the initial improvements in nitrogen utilization last across multiple production cycles or during key physiological transitions.

In addition, the specific metabolic pathways activated by BH replacement, especially those linked to the breakdown of β-glucans and the synthesis of volatile fatty acids, are still not fully understood. Future studies should use advanced methods such as shotgun metagenomics to move from basic taxonomic identification to true functional profiling.

Furthermore, the exact mechanisms behind the physiological changes seen at higher levels of BH inclusion remain unclear in our dataset. While we observed an increase in MDA levels and a decrease in CAT activity, our study did not measure intracellular reactive oxygen species or mitochondrial function in ruminal epithelial cells. Therefore, the hypothesis that rapid volatile fatty acid production causes metabolic oxidative stress still needs further validation.

## 5. Conclusions

Overall, substituting traditional forage with barley hay (BH) modulates ruminal fermentation and systemic immunity by enriching beneficial fiber-degrading bacteria. Although higher BH inclusion levels induced alterations in the antioxidant system, the 100% BH substitution (BH100) achieved an optimal physiological balance among the treatments. Specifically, BH100 proved superior by maximizing acetate and immunoglobulin production without causing severe oxidative detriment. Furthermore, BH inclusion facilitated a shift toward beneficial microbial taxa without negatively impacting alpha diversity. Consequently, the complete replacement of traditional forage with BH serves as a viable and promising nutritional strategy to optimize ruminal fermentation and enhance immunity in meat sheep, provided that the animals’ antioxidant status is appropriately monitored.

## Figures and Tables

**Table 1:** Ingredient composition and nutrient levels of the experimental diets (DM, %).

Items ^1^	Treatments ^2^				
	CON	BH25	BH50	BH75	BH100
**Ingredients**					
Barley Hay		10.30	20.60	30.90	41.19
*Leymus chinensis*	10.21	7.66	5.10	2.55	
Corn Stover	30.98	22.23	15.49	7.75	
Commercial Diet	38.47	39.01	39.55	40.09	40.63
Corn Silage	19.32	18.78	18.24	17.69	17.16
Limestone	0.12	0.12	0.12	0.12	0.12
NaCl	0.30	0.30	0.30	0.30	0.30
Sodium Bicarbonate	0.20	0.20	0.20	0.20	0.20
Premix	0.40	0.40	0.40	0.40	0.40
Total	100.00	100.00	100.00	100.00	100.00
**Nutrient Levels**					
Metabolic Energy (MJ, kg)	14.91	14.86	14.56	14.37	14.34
CP	13.62	13.79	14.05	14.21	14.67
ADF	23.86	24.43	26.01	26.58	27.15
NDF	43.87	45.24	46.61	47.98	49.35
Ca	1.18	1.19	1.21	1.22	1.24
P	0.61	0.60	0.59	0.58	0.57

^1^ Commercial diet, the basal diet used in this study was an on-farm custom-mixed diet routinely fed to meat sheep. The premix provided the following per kg of food: 3100 IU of VA; 1300 IU of VD3; 22 IU of VE; 24 mg of Fe; 8 mg of Cu; 0.1 mg of Co; 30 mg of Mn; 0.35 mg of Se; 30 mg of Zn; 0.45 mg of I. Metabolizable energy values were determined following the calculation method of Ding et al. [23], while the other nutrient levels are measured values. CP—crude protein; ADF—acid detergent fiber; NDF—neutral detergent fiber; Ca—calcium; P—phosphorus. ^2^ BH25, BH50, BH75, and BH100 represent the replacement of 25%, 50%, 75%, and 100% of the basal roughage (a mixture of corn stover and *Leymus chinensis*) with barley green hay, respectively.

**Table 2:** Nutrient profiles of the forage sources used in the experimental diets (DM, %).

Nutrient Levels ^1^	Barley Hey	Corn Stover	*Leymus chinensis*
Metabolic energy (MJ/kg)	12.77	13.31	13.88
CP	8.50	4.20	8.44
ADF	34.30	24.93	29.07
NDF	54.78	60.01	57.69
Ca	0.33	0.28	0.35
P	0.23	0.13	0.15

^1^ Metabolizable energy values were determined following the calculation method of Ding et al. [23], while the other nutrient levels are measured values. CP—crude protein; ADF—acid detergent fiber; NDF—neutral detergent fiber; Ca—calcium; P—phosphorus.

**Table 3:** Effects of replacing dietary forage with barley hay on ruminal fermentation characteristics in lambs.

Items ^1^	Treatments ^2^					SEM	*p*-Value
	CON	BH25	BH50	BH75	BH100		
pH	6.65	6.68	6.70	6.82	6.72	0.15	0.938
Acetate, mmol/L	15.40 ^b^	29.06 ^a^	34.28 ^a^	29.89 ^a^	36.37 ^a^	4.16	0.014
Propionate, mmol/L	7.87	7.30	7.36	4.49	6.39	0.85	0.068
Butyrate, mmol/L	5.98	3.44	5.57	4.34	3.83	0.73	0.087
Isobutyrate, mmol/L	0.58	0.50	0.58	0.63	0.64	0.09	0.853
Valerate, mmol/L	0.54	0.48	0.54	0.58	0.51	0.07	0.897
Isovalerate, mmol/L	1.11	0.87	1.05	1.08	1.07	0.14	0.781
A:P	2.13 ^b^	4.40 ^ab^	4.65 ^ab^	6.66 ^a^	6.37 ^a^	0.89	0.010
TVFA, mmol/L	31.47	41.65	49.38	41.01	48.81	4.92	0.100
NH_3_-N, mg/dL	2.98 ^a^	2.63 ^abc^	2.33 ^c^	2.84 ^ab^	2.44 ^bc^	0.15	0.033

^1^ A:P—the acetate to propionate ratio; TVFA—total volatile fatty acids; NH_3_-N—ammonia nitrogen. ^2^ BH25, BH50, BH75, and BH100 indicate the substitution of dietary forage with barley hay at levels of 25%, 50%, 75%, and 100%, respectively. Means with different superscript letters differ significantly (*p* < 0.05) or highly significantly (*p* < 0.01).

**Table 4:** Results of alpha-diversity indices among treatment groups.

Estimators	Treatments ^1^					Adjusted *p*-Value
	CON	BH25	BH50	BH75	BH100	
Ace	1493.44	1673.06	1768.95	1608.75	1405.27	0.239
Chao	1465.0651	1637.72	1726.15	1572.16	1378.36	0.218
Sobs	1443.00	1611.17	1693.17	1540.17	1356.83	0.207
Shannon	5.89	5.95	6.25	5.88	5.79	0.153
Coverage	1.00	1.00	1.00	1.00	1.00	0.893
Simpson	0.01	0.02	0.01	0.02	0.01	0.545

^1^ BH25, BH50, BH75, and BH100 indicate the substitution of dietary forage with barley hay at levels of 25%, 50%, 75%, and 100%, respectively.

**Table 5:** Effects of replacing dietary forage with barley hay on serum immune parameters in lambs.

Items ^1^	Treatments ^2^					SEM	*p*-Value
	CON	BH25	BH50	BH75	BH100		
**day 30**							
IgA, μg/mL	91.83 ^c^	94.23 ^bc^	109.49 ^a^	113.41 ^a^	101.07 ^b^	1.76	<0.001
IgG, μg/mL	1277.10 ^b^	1279.02 ^b^	1392.59 ^a^	1395.98 ^a^	1311.30 ^b^	22.99	<0.001
IgM, μg/mL	12.52	12.71	12.48	12.98	12.37	0.20	0.224
IL-1β, ng/L	116.06	117.43	115.10	121.41	120.38	1.77	0.079
IL-4, pg/mL	132.15 ^a^	130.09 ^a^	129.88 ^a^	124.32 ^b^	122.16 ^b^	1.84	0.003
IL-6, ng/L	102.95 ^c^	132.64 ^b^	145.55 ^a^	149.93 ^a^	127.60 ^b^	0.77	<0.001
IL-10, ng/L	203.27 ^a^	200.11 ^a^	202.21 ^a^	193.17 ^a^	182.60 ^b^	3.46	0.001
TNF-α, ng/L	1080.26	1111.43	1102.49	1109.29	1067.03	20.81	0.490
**day 60**							
IgA, μg/mL	93.04 ^d^	94.95 ^cd^	107.38 ^b^	114.63 ^a^	99.68 ^c^	1.65	<0.001
IgG, μg/mL	1271.97 ^b^	1288.64 ^b^	1364.36 ^a^	1360.68 ^a^	1288.64 ^ab^	21.56	0.013
IgM, μg/mL	12.22	12.61	12.27	12.61	12.10	0.15	0.075
IL-1β, ng/L	117.75	117.67	112.47	117.54	119.09	2.97	0.575
IL-4, pg/mL	132.36 ^a^	131.66 ^a^	132.75 ^a^	123.57 ^b^	122.70 ^b^	1.66	<0.001
IL-6, ng/L	103.17 ^d^	135.08 ^c^	141.51 ^b^	148.93 ^a^	130.54 ^c^	0.89	<0.001
IL-10, ng/L	203.77 ^a^	199.35 ^a^	204.39 ^a^	186.31 ^b^	184.20 ^b^	2.70	<0.001
TNF-α, ng/L	1020.59 ^b^	1114.39 ^a^	1115.52 ^a^	1105.74 ^a^	1032.93 ^b^	18.29	<0.001

^1^ IgA—immunoglobulin A; IgG—immunoglobulin G; IgM—immunoglobulin M; IL-1β—interleukin-1β; IL-4—interleukin-4; IL-6—interleukin-6; IL-10—interleukin-10; TNF-α—tumor necrosis factor α. ^2^ BH25, BH50, BH75, and BH100 indicate the substitution of dietary forage with barley hay at levels of 25%, 50%, 75%, and 100%, respectively. Means with different superscript letters differ significantly (*p* < 0.05) or highly significantly (*p* < 0.01).

**Table 6:** Effects of replacing dietary forage with barley hay on serum antioxidant parameters in lambs.

Items ^1^	Treatments ^2^					SEM	*p*-Value
	CON	BH25	BH50	BH75	BH100		
**day 30**							
T-AOC, U/mL	7.83	7.60	7.64	7.69	7.71	0.10	0.559
SOD, pg/mL	233.22	224.11	226.87	213.62	222.22	4.44	0.062
CAT, ng/L	210.14 ^a^	190.42 ^b^	192.04 ^b^	197.58 ^b^	190.89 ^b^	3.49	0.002
GSH-Px, pmol/mL	63.16	61.02	61.97	60.00	60.89	1.12	0.350
MDA, nmol/mL	10.64 ^b^	11.14 ^a^	11.32 ^a^	11.43 ^a^	11.09 ^a^	0.34	0.006
**day 60**							
T-AOC, U/mL	7.83	7.57	7.65	7.48	7.67	0.11	0.271
SOD, pg/mL	232.81	219.05	226.77	217.49	224.11	3.92	0.070
CAT, ng/L	219.85 ^a^	186.66 ^c^	191.26 ^bc^	187.83 ^bc^	198.70 ^b^	3.72	<0.001
GSH-Px, pmol/mL	62.30	59.78	62.07	59.08	60.30	1.04	0.148
MDA, nmol/mL	10.89 ^b^	10.84 ^b^	11.39 ^ab^	11.68 ^a^	11.15 ^ab^	0.46	0.020

^1^ T-AOC—total antioxidant capacity; SOD—superoxide dismutase; CAT—catalase; GSH-Px—glutathione peroxidase; MDA—malondialdehyde. ^2^ BH25, BH50, BH75, and BH100 indicate the substitution of dietary forage with barley hay at levels of 25%, 50%, 75%, and 100%, respectively. Means with different superscript letters differ significantly (*p* < 0.05) or highly significantly (*p* < 0.01).

## Data Availability

The original contributions presented in this study are included in the article. Further inquiries can be directed to the corresponding authors.
